# Novel *MASP1* mutations are associated with an expanded phenotype in 3MC1 syndrome

**DOI:** 10.1186/s13023-015-0345-3

**Published:** 2015-09-30

**Authors:** Tahir Atik, Asuman Koparir, Guney Bademci, Joseph Foster, Umut Altunoglu, Gül Yesiltepe Mutlu, Sarah Bowdin, Nursel Elcioglu, Gulsen A. Tayfun, Sevinc Sahin Atik, Mustafa Ozen, Ferda Ozkinay, Yasemin Alanay, Hulya Kayserili, Steffen Thiel, Mustafa Tekin

**Affiliations:** Dr. John T. Macdonald Foundation Department of Human Genetics and John P. Hussman Institute for Human Genomics, Miller School of Medicine, University of Miami, 1501 NW 10th Avenue, BRB-610 M-860, Miami, FL 33136 USA; Division of Genetics, Department of Pediatrics, Ege University School of Medicine, Izmir, Turkey; Department of Medical Genetics, Cerrahpasa Medical School, Istanbul University, Istanbul, Turkey; Department of Medical Genetics, Istanbul Medical School, Istanbul University, Istanbul, Turkey; Division of Pediatric Endocrinology and Diabetes, Kocaeli University School of Medicine, Kocaeli, Turkey; Division of Clinical and Metabolic Genetics, Department of Paediatrics, The Hospital for Sick Children, Toronto, Ontario Canada; Division of Genetics, Department of Pediatrics, Marmara University School of Medicine, Istanbul, Turkey; Department of Ophthalmology, Ataturk Teaching and Research Hospital, Katip Celebi University, Izmir, Turkey; Department of Medical Genetics/Molecular Biology and Genetics Biruni University, Istanbul, Turkey; Division of Genetics, Department of Pediatrics, Acibadem University Medical Faculty, Istanbul, Turkey; Medical Genetics Department, Koç University School of Medicine, Istanbul, Turkey; Department of Biomedicine, Aarhus University, Aarhus, Denmark

**Keywords:** 3MC syndrome, Complement, Lectin pathway, MASP-1, MASP-3

## Abstract

**Background:**

3MC1 syndrome is a rare autosomal recessive disorder characterized by intellectual disability, short stature and distinct craniofacial, umbilical, and sacral anomalies. Five mutations in *MASP1*, encoding lectin complement pathway enzymes MASP-1 and MASP-3, have thus far been reported to cause 3MC1 syndrome. Only one previously reported mutation affects both MASP-1 and MASP-3, while the other mutations affect only MASP-3.

**Methods:**

We evaluated six unrelated individuals with 3MC1 syndrome and performed Sanger sequencing for all coding exons of *MASP1.* We also measured complement lectin and alternative pathway activities in an affected individual’s serum.

**Results:**

We found two novel splice site mutations, c.1012-2A > G in one and c.891 + 1G > T in two probands, and three novel missense mutations, c.1451G > A (p.G484E), c.1657G > A (p.D553N), and c.1987G > T (p.D663Y). Missense mutations affect only MASP-3, while splice site mutations affect both MASP-1 and MASP-3. In a proband who is homozygous for c.891 + 1G > T, we detected a total lack of lectin complement pathway activity and a 2.5-fold lower alternative pathway activity. The phenotype observed in patients whose both MASP-1 and MASP-3 are affected and in those whose only MASP-3 is affected does not appear to be different. We observed structural brain abnormalities, neonatal tooth, a vascular anomaly and a solid lesion in liver as novel phenotypic features of 3MC1 syndrome.

**Conclusion:**

Novel mutations and additional phenotypic features expand the genotypic and phenotypic spectrum of 3MC1 syndrome. Although patients with MASP-1 dysfunction in addition to disrupted MASP-3 have an altered complement system, their disease phenotype is not different from those having only MASP-3 dysfunction.

**Electronic supplementary material:**

The online version of this article (doi:10.1186/s13023-015-0345-3) contains supplementary material, which is available to authorized users.

## Background

3MC syndrome (MIM 257920;265050;248340) is characterized by distinctive facial features including hypertelorism, blepharophimosis, blepharoptosis, highly arched eyebrows, and cleft lip/palate, postnatal growth retardation, variable degree of developmental delay or intellectual disability, and hearing loss. Craniosynostosis, radioulnar synostosis, genital and vesicorenal anomalies, caudal appendage, and umbilical hernia/ omphalocele or periumbilical depression are also often seen in this syndrome. 3MC syndrome has been named by lumping Michels, Malpuech, Mingarelli and Carnevale syndromes, which were originally reported to be separate entities with overlapping features [1, 2].

**Fig. 1:**
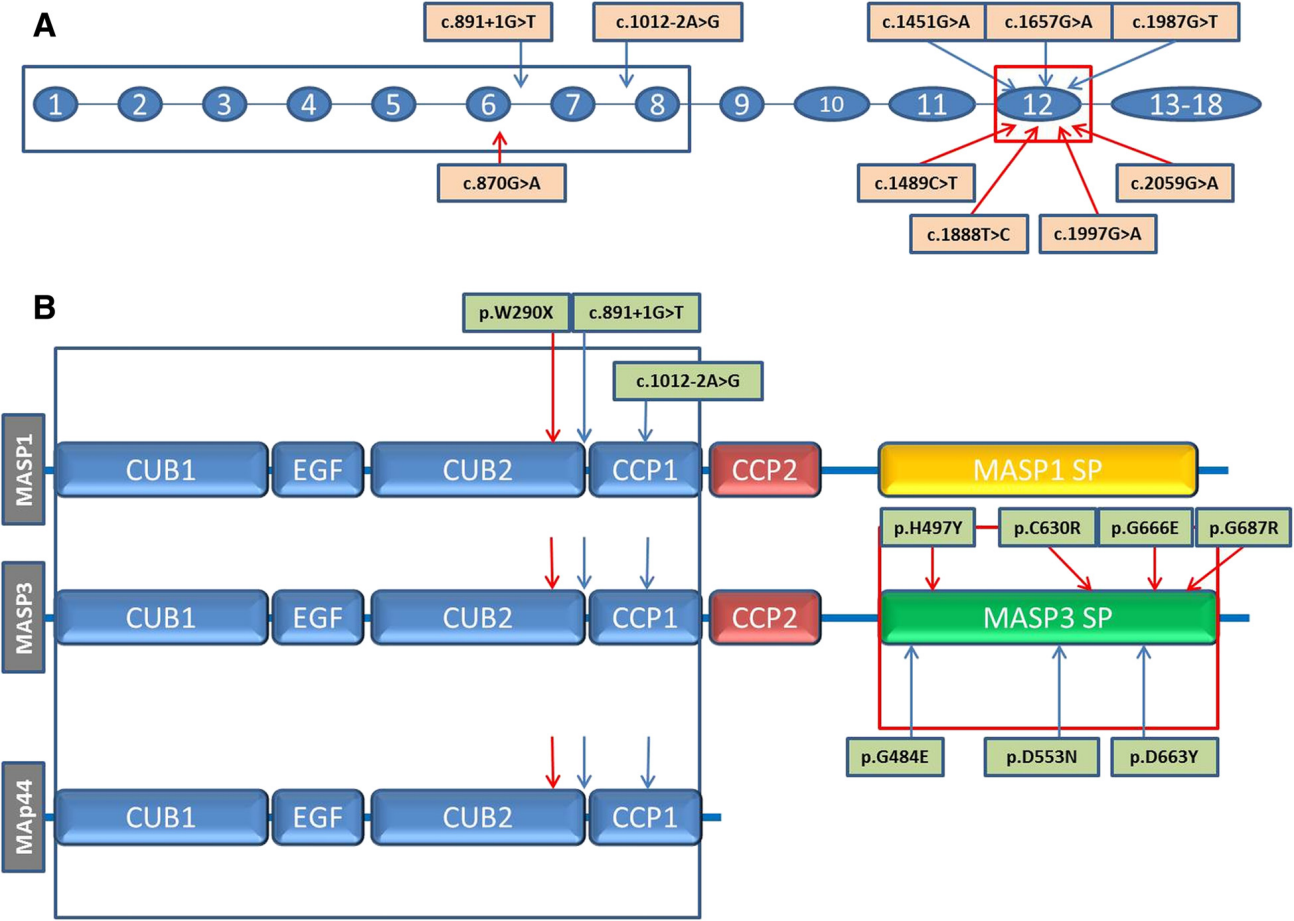
**a** Overview of *MASP1* mutations. Exons and introns are indicated by circles with numbers and thin lines, respectively. The localization of mutations identified in this study is shown with blue arrows; those reported by Sirmaci et al.[3] and Rooryck et al. [4] are shown with red arrows; **b** Overview of the resulting protein structures of the three mRNA sequences produced from *MASP1.* In protein domains of these different transcripts, the blue arrows and red arrows show the mutations presented in this study and reported previously, respectively [3, 4]

Mutations in two genes, *MASP1* (MIM 600521) and *COLEC11* (MIM 612502), which both encode proteins playing important roles in the lectin complement pathway, were found to be responsible for 3MC syndrome [3, 4]. While there is no recognized phenotypic difference, OMIM classifies individuals having mutations in *MASP1* and *COLEC11* under 3MC1 (MIM 257920) and 3MC2 (MIM 265050) syndromes, respectively. The *MASP1* gene is located on chromosome 3q27-28 and gives rise to three alternative splice products playing roles in the lectin pathway of the innate immune system: MASP-1, MASP-3, and MAp44 [5–7]. The gene is composed of 18 exons; the three alternative splice products share the same first eight exons encoding the first four domains of the proteins. The serine protease (SP) domains of MASP-1 and MASP-3 are encoded by separate exons, whereas no SP domain is present in MAp44 (Fig. 1). The SP domain of MASP-1 is encoded by six separate exons (13–18), while MASP-3 is encoded by a single exon (12) [8–10]. In this study, we report on six previously unreported children with 3MC1 syndrome and the results of their molecular investigations.

## Methods

Six unrelated probands clinically diagnosed with 3MC syndrome and their parents were included in this study. Written informed consent was obtained from all participants. The study was approved by the Institutional Review Board at the University of Miami.

### Molecular studies

Genomic DNA was extracted from peripheral leukocytes according to standard protocols. Sanger sequencing for all coding exons of the *MASP1* gene was performed on all patients and mutated exons were analyzed for cosegregation in their unaffected parents (primer sequences are available on request). A touchdown PCR protocol was used to amplify target DNA region. The resulting amplicons were cleaned up using Sephadex G-25 beads (GE Healthcare). An ABI PRISM 3730 DNA analyzer (Applied Biosystems) and Big Dye Terminator Cycle Sequencing V3.1 Ready Reaction Kit (Life Technologies) were used to elucidate the DNA sequence. Variants were named according to NM_139125.3.

To evaluate the implication of splice site mutations, we used The Berkeley Drosophila Genome Project (PDGP) (http://www.fruitfly.org/) as a splice site prediction program. Missense variants were analyzed for having a pathogenic prediction score at least in two of the following tools: PolyPhen2, SIFT, and MutationTaster. PhyloP and GERP scores were used to evaluate the levels of conservation of the regions in which the mutations were located [11–16].

### Functional studies

The levels of MASP-1, MASP-3 and MAp44 were measured by sandwich type immunoassays by the use of europium labelled detection reagent and measurement by time-resolved fluorescence as previously described in detail [17].

For measurement of the activity of the alternative pathway of complement, we followed a previously published procedure for the estimation of the ability of a sample to mediate lysis of rabbit erythrocytes, in a buffer inhibiting the classical and the lectin pathway of complement as described [17]. Serum samples were diluted in gelatin-veronal buffer with magnesium and EGTA (a negative control containing EDTA buffer was also included). Twenty microliters of each dilution was mixed with 10 μl 6 % (v/v) rabbit erythrocytes in the same buffer and the mixture incubated for 2 h at 37 °C after which the lysis reactions were stopped by the addition of EDTA, and the mixture centrifuged followed by reading the absorbance at 405 nm of the supernatant.

The estimation of functional activity of the lectin pathway followed a previously published procedure that measures the ability of a serum to deposit C4 fragments onto a mannan surface [17]. Since the serum from patient 3 contains a low mannan binding lectin (MBL) level (i.e. is MBL deficient) it was reconstituted to physiological MBL level (2 μg MBL/ml) with recombinant MBL. The normal serum we used for comparison was also MBL deficient and was similarly added MBL to 2 μg/ml. Sera were diluted in calcium containing buffer and added to mannan-coated microtiter wells. After incubation overnight at 4 °C the wells were washed and now purified human C4 was added. After incubation at 37 °C for 1 h, during which period C4b is deposited on the surface, the wells were washed, and biotinylated anti-C4 antibody was added. After incubation and wash europium-labeled streptavidin was added. After incubation and wash an enhancement solution was added, followed by reading of time-resolved fluorescence.

## Results

### Clinical features

**Fig. 2:**
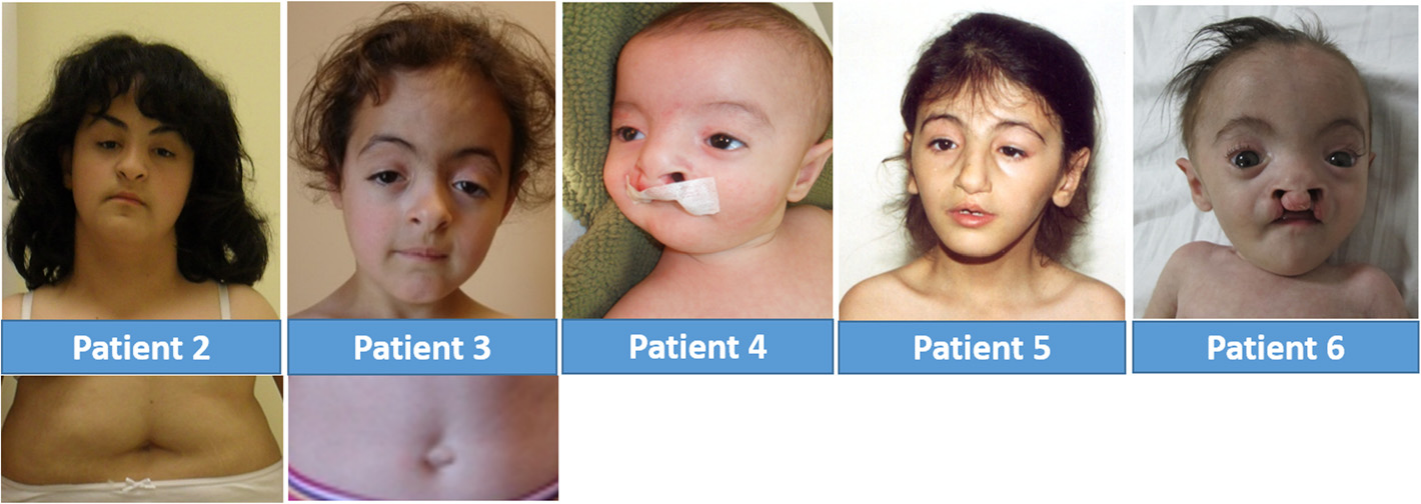
Photographs showing distinctive phenotypic features in Patients 2-6

The ages of probands ranged from 4 months to 22 years. There were three males and three females. All six patients were born at term. Their weights, heights and head circumferences were in normal limits at birth but three of them showed postnatal growth retardation. Pedigrees, photographs and clinical features of all patients are shown in Table 1, Fig. 2 and Additional file 1: Figure S1.

**Table 1:** A summary of *MASP1* mutations and phenotypic features of affected individuals

	Patients described in the present report						Previously reported patients									
Patient no (P)	P2	P3	P4	P1	P5	P6	II-101^3^	MC6_1^2,4^	MC6_2^2,4^	MC7_1^4,22^	MC7_2 ^4,22^	I-101^3^	I-102^3^	MC3_1^4^	MC5_1^4^	MC5_2^4^
Mutation (cDNA)	c.1012-2A > G	c.891 + 1G > T	c.891 + 1G > T	c.1451G > A	c.1657G > A	c.1987G > T	c.870G > A	c.1997G > A	c.1997G > A	c.1997G > A	c.1997G > A	c.2059G > A	c.2059G > A	c.1489C > T	c.1888 T > C	c.1888 T > C
Mutation (protein)	splice	splice	splice	p.G484E	p.D553N	p.D663Y	p.W290X	p.G666E	p.G666E	p.G666E	p.G666E	p.G687R	p.G687R	p.H497Y	p.C630R	p.C630R
Localization	Intron 7	Intron 6	Intron 6	Exon 12	Exon 12	Exon 12	Exon 6	Exon 12	Exon 12	Exon 12	Exon 12	Exon 12	Exon 12	Exon 12	Exon 12	Exon12
Origin	Turkey	Turkey	Turkey	Pakistan	Turkey	Syria	Turkey	Brazil	Brazil	Brazil	Brazil	Turkey	Turkey	Greece	Italy	Italy
Consanguinity	+	+	+	+	+	+	+	-	-	+	+	+	+	+	-	-
Age	15 years	6.5 years	4 months	4.5 years	22 years	6 months	9 years	20 years	14 years	23 years	17 years	15 years	10 years	NA	NA	NA
Gender	F	F	M	M	F	M	F	F	M	M	M	F	F	M	M	M
Short Stature	-	+	-	+	+^#8^	+	-	-	-	+	+	-	-	-	+	+
Intellectual disability/developmental delay	-	-	NA	+	-	NA	+	-	-	-	-	+	+	-	-	-
Craniosynostosis/Skull asymmetry	NA	-	+	NA	+	-	NA	-	-	-	-	NA	NA	+	-	-
Arched eyebrows	+	+	+	+	+	+	+	+	+	+	+	+	+	+	+	+
Hypertelorism	+	+	+	+	+	+	-	+	+	+	+	+	+	+	+	+
Blepharoptosis	+	+	+	+	+	+	+	+	+	+	+	+	+	+	+	+
Downslanting palpebral fissures	+	+	+	+	+	+	+	+	+	+	+	+	+	+	+	+
Anterior chamber anomalies	-	+^#3^	-	+^#2^	-	-	-	+	-	-	-	-	-	-	-	-
Hearing loss	-	-	+	+	+	NA	-	+	+	+	+	+	+	-	+	+
Cleft lip/palate	-	-	+	+	+	+	-	-	-	+	+	+	+	+	+	+
Periumbilical depression	+	+	+	NA	+	-	+	+	-	+	+	+	+	-	-	-
Omphalocele/Umblical hernia	-	-	-	+	+	-	-	+	+	-	-	-	-	-	-	-
Limitation of elbow movements	-	-	-	-	-	-	-	+	-	+	+	+	-	-	-	-
Genital anomalies	-	-	+^#5^	-	-	+^#9^	-	-	+	+	+	-	-	-	+	+
Vesicorenal anomalies	-	+^#4^	+^#6^	-	-	+^#10^	+	-	-	-	+	+	-	+	-	-
Caudal appendage and/or spina bifida	+	-	+	SPO^#1^	+^#7^	+	+	+	+	+	-	+	+	-	+	+
Cardiac	-	PDA	PDA	ASD, PDA	-	ASD, PDA	PDA	-	-	-	-	-	-	-	-	-
Structural anomaly in Cranial/Spinal MRI	NA	NA	-	+^#1^	+^#7^	NA	NA	NA	NA	NA	NA	NA	NA	NA	NA	NA
Other			Vascular lesion in liver	Neonatal tooth, left clubfoot	Accessory nipple on the left, bilateral fifth finger campto-clinodactyly	Solid lesion in liver^#11^										

NA: not available; ASD: atrial septal defect; PDA: patent ductus arteriosus; SPO: Spina bifida occulta; mutations affecting all protein products of *MASP1* are grayed

#1: Absent olfactory bulbs, small pituitary gland, small thin corpus callosum and severe narrowing of the foramen magnum in cranial MRI, and adequate alignment with conus medullaris at L3, and a 9 mm intrathecal cyst in the distal sac in spinal MRI; #2: Left sided Peter’s anomaly; #3: Bilateral leukoma; #4: Cross renal ectopia; #5: Ambiquous genitalia; #6: Horseshoe kidney; #7: Spinal MRI at 2 years of age revealed a spina bifida between L3 and the sacrum, lumbosacral dural sac ectasia, an intradural dermoid tethering of the cord between L2 and S4, with a thickened filum terminale at L2. She underwent an operation for excision of the cystic tumor, tethered cord, and bone repair; #8: Growth hormone treatment was administered for the patient until 17 years of age; #9: Cliteromegaly of 1.5 cm, anterior ectopic anus, prominent coccyx with a sacral pit, and a presacral capillary malformation of 2 × 3 cm were observed; #10: Left renal agenesis; #11: Abdominal ultrasound showed a heterogeneous hypoechoic solid lesion of 38 × 24 mm, containing serpiginous tubular structures, in the anterior segment of the right lobe of the liver

Patients 1 and 6 showed a more severe clinical presentation and poor prognosis.

In patient 1, bilateral cleft lip and palate, left clubfoot, neonatal tooth, omphalocele and a sacral dimple with a hairy patch were noted at birth. Early gross motor developmental milestones were moderately delayed, however at the age of 2 years, the patient developed meningitis and subsequently his developmental milestone acquisition slowed or plateaued in all spheres. At his current age of 4 years 6 months, he is able to walk with support but unable to feed himself. He makes no eye contact and has not developed alternative means of communication, in part due to profound hearing loss. He remains G-tube fed, and his weight and height are both 5–6 SD below the mean. On ophthalmologic examination, he was noted to have left sided Peter’s anomaly, in addition to hypermetropia. Brain MRI indicated absent olfactory bulbs, small pituitary gland, small thin corpus callosum and severe narrowing of the foramen magnum. A spinal MRI also revealed adequate alignment with conus medullaris at L3, and a 9 mm intrathecal cyst in the distal sac. A secundum atrial septal defect (ASD), patent ductus arteriosus (PDA) and pulmonary hypertension were detected on echocardiogram.

Patient 6 was noticed to have bilateral cleft lip and palate, and clitoromegaly at birth. Echocardiography revealed a secundum ASD with a wide PDA. A diagnostic work up including a head ultrasound, serum electrolytes, detailed biochemical screening such as liver enzymes, and renal function tests, were all within normal limits. Abdominal ultrasound showed a heterogeneous hypoechoic solid lesion of 38 × 24 mm, containing serpiginous tubular structures, in the anterior segment of the right lobe of the liver; the left renal agenesis was also observed. On examination at 6 months of age, she had a weight of 3200 g (<3^th^ centile), height of 58 cm (<3^th^ centile), and head circumference of 39 cm (<3^th^ centile). Anterior fontanelle was 3 × 4 cm, and posterior fontanelle was still palpable as 1 × 1 cm. In addition to typical facial features of 3MC syndrome, clitoromegaly of 1.5 cm, anterior ectopic anus, prominent coccyx with a sacral pit, and a presacral capillary malformation of 2 × 3 cm were observed. She was hypotonic with normal deep tendon reflexes. Head control was not achieved. Although cell culture from peripheral blood lymphocytes was unsuccessful, interphase FISH study with centromeric probes showed two signals for X chromosome and none for Y. 17-hydroxyprogesteron, androstenedione, cortisol levels, and plasma renin activity were normal. The patient underwent a successful PDA ligation operation, but unfortunately died 2 months later due to pneumonia.

Patient 4 was followed up until age 4.5 months and did not present with significant clinical problems other than features described in Table 1.

In three older patients (patients 2, 3, and 5) long term follow ups were available. Patient 2 showed normal developmental milestones from birth to 15 years old and did not experience any significant health problem.

Patient 3 presented with recurrent bronchiolitis episodes and her weights, heights and head circumferences were in lower limits of normal at the age of 6. She had normal cognitive functions.

Patient 5 underwent an operation for excision of the cystic tumor, tethered cord, and bone repair because of her lumbosacral anomalies at the age of 2. Her neuromotor development was on target except for a mild speech delay. Audiometric evaluation revealed moderate conductive hearing loss on the right, due to chronic otitis media. At 9 years and 2 months of age, her height was 122.5 cm (3^rd^ centile), weight was 21.7 kg (3–10^th^ centile), and head circumference was 52 cm (25–50^th^ centile). A WISC-R IQ score was 98. At age 12, she was evaluated for short stature, and was found to have growth hormone deficiency. Growth hormone treatment was administered until age 17. At 22 years of age, her height was 153 cm (3–10^th^ centile), weight was 40 kg (<3^th^ centile), and head circumference was 54.5 cm (3–10^th^ centile). She was working as a clerk in a state institution, and did not report additional health problems.

### Molecular findings

We detected five novel mutations in the *MASP1* gene (Fig. 1 and Additional file 1: Figure S2). One mutation, affecting the splice donor site in intron 6 (c.891 + 1G > T), was found in two probands (Patient 3 and 4). Another splice site mutation was found in the acceptor site of intron 8 (c.1012-2A > G)(Patient 2). As expected from their locations at consensus splice sites, both c.891 + 1G > T and c.1012-2A > G were found to be in highly conserved regions: PhyloP scores are 4.88 for both and GERP scores are 5.73 and 5.7, respectively. The c. 891 + 1G > T is predicted to lead to the skipping of exon 6 affecting both MASP-1 and MASP-3 proteins. The second splice site mutation, c.1012-2A > G, is predicted to lead to replace the acceptor site of exon 8 with a new site located 239 bp upstream of that point.

The other three mutations were missense (p.G484E in Patient 1, p.D553N in Patient 5, and p.D663Y in Patient 6), located in exon 12. None of these was present as homozygous variation in the EVS (http://evs.gs.washington.edu/EVS/) and ExAC databases (http://exac.broadinstitute.org/). All missense mutations were predicted to be disease causing when tested using *in silico* prediction tools (Polyphen, MutationTaster, and SIFT) and were located in very conserved regions. According to an *in silico* protein structure analysis (HOPE) [18], of these missense mutations, p.G484E and p.D663Y were located in part of 4 different interpro domains (Peptidase S1; Peptidase S1A, chymotrypsin-type; trypsin-like cysteine/serine peptidase; Peptidase S1A, complement C1r/C1S/mannan-binding) which are important for the main activity of the protein. The mutations in these regions can disturb the core structure of the protein and thereby affect the catalytic activity. The other missense mutation identified in this study, p.D553N, is located in the active site and a mutation at this site is, always, expected to disturb the function of the protein [19]. All variants co-segregated with the phenotype as an autosomal recessive disease in available family members (parental samples were not available for patient 1).

### Functional characterization of complement pathways

In the present report we tested the serum levels of MASP-1, MASP-3 and MAp44 of patient 3, who is homozygous for c.891 + 1G > T. For all three proteins we did not detect a signal. We further found that the patient was MBL deficient, i.e. MBL level was below 100 ng/ml (this occurs in approximately 10 % of the general population).

**Fig. 3:**
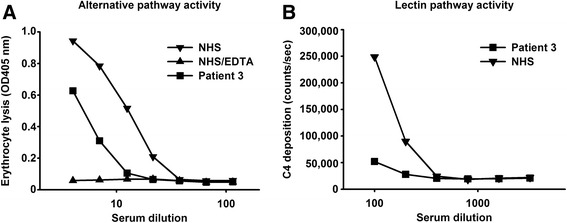
Assessment of alternative and lectin pathway activity of patient 3 serum. **a** Rabbit erythrocyte lysis via the alternative pathway by normal human serum (NHS) and patient 3 serum and as a negative control by NHS diluted in EDTA buffer (NHS-EDTA). The x-axis gives the dilution of the sera tested whereas the y-axis gives the degree of lysis of the erythrocytes as measured by absorption at 405 nm of the supernatant after incubation of sera and the cells. Representative of two experiments with similar results. Error bars for duplicates are at a size causing them to be covered by the symbols; **b** C4 deposition in mannan-coated wells. Dilutions of serum from patient 3 and from a normal human serum (NHS) were incubated in the wells. Since both sera are MBL deficient they were reconstituted with recombinant MBL to the same physiological level of 2 μg/ml. The x-axis give the dilution of the sera and the y-axis gives the C4b deposition in counts per second as detected by anti-C4 antibodies. Representative of two experiments with similar results. Error bars for duplicates are at a size causing them to be covered by the symbols

In the literature there have been reports on the involvement of MASP-1 and MASP-3 on the alternative pathway activity of serum [20, 21]. We have previously seen that a patient devoid of MASP-1 and MASP-3 still contained the ability to lyse rabbit erythrocytes, which is traditionally used for testing the activity of the alternative pathway [17]. We found that patient 3 from the present report, who has no MASP-1 and MASP-3 still had the ability to initiate the alternative pathway (Fig. 3a). The activity was 2.5-fold lower than the sample from a normal healthy individual tested at the same time and the negative control including EDTA in the buffer gave only background levels. The function of MASP-1 is described as activating MASP-2, which then cleaves C4 and thus generating the C4b form, which may covalently bind to nearby surfaces. When we tested the ability of patient 3 serum to deposit C4b onto a mannan surface, we found very low C4b deposition as compared to a normal human serum (Fig. 3b). This assay is dependent on the MBL concentration of the serum tested, but in this case we were lucky that patient 3 is MBL deficient and we could thus control the amount of MBL by adding a known amount of MBL to the serum. We then picked a MBL deficient serum from a blood donor cohort and added the same amount of MBL for comparison. We furthermore examined whether the MBL pathway would be affected by reconstitution of the patient serum with recombinant MASP-1 and found this to be the case, whereas no increase of MBL pathway activity was found in the normal serum when rMASP-1 was added (not shown).

## Discussion

The MBL-associated serine proteases (MASPs), MASP-1, MASP-2 and MASP-3, are proteins playing crucial roles in the lectin complement pathway [17]. In addition to their functions in the complement system, some unexpected roles of these proteins have been identified in two recent studies. Sirmaci et al. showed that homozygous *MASP1* mutations cause 3MC syndrome in two unrelated families [3]. While one missense mutation was in the exon encoding the SP domain of the protein MASP-3, the other mutation was a nonsense variant in the exon encoding a domain (CUB2) shared by MASP-1, MASP-3, and MAp44 proteins [3]. Rooryck and colleagues described three additional missense mutations affecting the same domain of MASP-3 protein in another four families [2, 4, 22].

Here we report five additional *MASP1* mutations. Three missense variants found in this study affect only the SP domain of MASP-3 and support the earlier observation that the disruption of MASP-3 with normal MASP-1 and MAp44 is sufficient to cause 3MC syndrome (Fig. 1). We also present three additional patients to the only one previously reported case with mutations disrupting all three protein products of *MASP1*, MASP-1, MASP-3 and MAp44 [3]. We previously published on functional effects of a 3MC1 patient homozygous for a c.870G > A (p.W290X) mutation in exon 6 of *MASP1* which gave rise to no MASP-1, MASP-3 or MAp44 in plasma of the patient. In the present report we describe that similarly we cannot measure any MASP-1, MASP-3 or MAp44 in the plasma of patient 3, who is homozygous for c.891 + 1G > T.

It was previously reported in mice that MASP-1 was the protease responsible for activating the proenzyme profactor D to the active enzyme Factor D, which is essential for activities of the alternative pathway of the complement system [20]. This finding was subsequently further extended by the same group reporting that MASP-3 could also cleave profactor D, and in addition, the complement factor B [21]. In a more recent paper Ruseva and colleagues demonstrated that MASP-1 and MASP-3 are not essential for activation of the alternative pathway *in vivo*, and they noted that this occurred in the absence of detectable mature factor D [23]. Further, it was found that in human serum from a 3MC patient with lack of MASP-1 and MASP-3 the proform of Factor D was dominating [24]. Comments on the interaction of other enzymes with profactor D giving rise to the alternative pathway activity that is still observed in the 3MC patient in the absence of MASP-1 or MASP-3 as well as an extended discussion on the assays used for the detection of activity of the alternative in the papers mentioned above have been published [25] and reported at meetings [26]. Rabbit erythrocytes activate the alternative complement pathway in human serum. We thus examined the ability of serum from patient 3 to lyse rabbit erythrocytes under conditions preventing activation of the classical and lectin pathways. We found alternative pathway activity being present although, as we previously reported [17], the activity does seem to be lower than the normal human serum we tested in parallel (Fig. 3a). Together, these results demonstrate that neither MASP-1 nor MASP-3 is crucial for alternative pathway activity in humans. With regards to the activity of the lectin pathway of the complement system, we tested the ability to deposit C4 fragments onto an MBL ligand surface (mannan). We have previously described that MBL/MASP-1 complexes activate MASP-2 in neighbor phenotype by testing MBL/MASP-2 complexes and that MASP-2 then starts to cleave C4, and others have also reported the need for MASP-1 to generate a fast and efficient lectin pathway activity [27, 28]. In accordance with these reports, we find that serum from patient 3 had very low lectin pathway activity when compared to a normal serum with same concentration of MBL (Fig. 3b). It would be possible to screen for a similar biochemical phenotype by initially testing for MASP-1 and MASP-3 levels. Testing for MASP-3 levels only may be sufficient as we have so far only detected MASP-3 deficiency in 3MC patients despite having tested numerous patient samples, i.e. more than 2500, representing many different diseases. To identify the patients who do have MASP-3 protein but a non-functional version of the serine protease domain (e.g. harboring some of the variants in the serine protease domain as given in Fig. 1) a substrate for MASP-3 is needed. Taking into account the reports discussed above this could possibly be profactor D, but more studies are needed before such tests can be developed as also other enzymes can cleave profactor D and a combination of catching MASP-3 in, e.g. microtiter wells combined with test for cleavage of profactor may be needed for this.

The 3MC1 syndrome is a rare disorder that affects many different tissues and organs (Table 1). Evaluation of phenotypic findings does not show any clear-cut difference between patients who have mutations affecting only MASP-3 and those who have mutations affecting all three *MASP1* protein products.

Although only one patient with *MASP1* mutation in the literature was reported to have anterior chamber anomalies [3, 4], Peter’s anomaly in one patient and bilateral leukoma in another patient was detected in our study. Previously, Guion-Almeida et al. reported a clinically diagnosed 3MC case with bilateral leukoma [29]. Molecular analysis was not performed in that patient. The presence of Peter’s anomaly and bilateral leukoma suggests that anterior chamber abnormalities are a genuine presentation of 3MC1 syndrome with *MASP1* mutations. A number of eye abnormalities, such as blepharophimosis, ptosis, lower eyelid eversion, anterior chamber abnormalities, have been reported in 3MC patients [1–3, 29]. The majority of them are eyelid developmental abnormalities (blepharophimosis, ptosis, lower eyelid eversion, etc.) Anterior chamber structures, (their abnormalities have rarely been reported in 3MC syndrome) include lens, iris, ciliary body and pupil. Certain anterior chamber anomalies such as leukoma and cataracts, are severe and required various surgical procedures. There has been no study investigating the effects of MASP proteins in eye development.

Other clinical features not reported in this syndrome until this study include a vascular anomaly and a solid lesion in liver, absent olfactory bulbs, small pituitary, neonatal tooth, and severe narrowing of foramen magnum. These additional features might be coincidental and need further studies for conclusion. In this study, MRI studies revealed cranial and spinal morphological abnormalities in patients 1 and 5 and a neurosurgical operation was performed on patient 5 for symptomatic spinal abnormality. We thus recommend imaging studies for detecting cranial and spinal anomalies in patients with 3MC1 syndrome.

3MC syndrome includes overlapping features of four syndromes, which were considered to be separate entities previously. Regarding the previous nosology, when we evaluate the patients presented here, Michels or Malpuech syndrome would be the most probable diagnosis for patients 1, 4 and 5, Carnavale syndrome for patient 2, Michels or Carnavale syndrome for patient 3.

Up to date, there have been 26 3MC patients reported, including our 6 patients, whose causative mutations are identified. Sixteen  of them had mutations in *MASP1*, 10 patients had mutations in *COLEC11*. While comparison of phenotypic features of patients with *COLEC11* and *MASP1* mutations would be speculative because of the limited number of patients, craniosynostosis and intellectual disability were reported in 27.2 and 28.5 % of patients with *MASP1* mutations, respectively, while they were observed in 60 and 70 % of patients with *COLEC11* mutations, respectively. Conversely, hearing loss, cleft lip/palate, genital anomalies, vesicorenal anomalies, and caudal appendages were reported to be more common in patients with *MASP1* mutations, compared to those with *COLEC11* mutations [3, 4, 22].

## Conclusion

Novel *MASP1* mutations and phenotypic features expand the genotypic and phenotypic spectrum of 3MC1 syndrome. Patients with disrupted MASP-1 and MASP-3 have altered complement system, while their phenotype doesn’t appear to be different from those with only disrupted MASP-3. We confirm that an alternative pathway activity is present in the MASP-1, MASP-3 and MAp44 deficient serum from a patient and that lectin pathway activity in serum from this patient is lacking.

## Additional file

Additional file 1: Figure S1. Pedigrees of the studied families. **Figure S2.** Electropherograms showing detected pathogenic variants. (DOCX 157 kb)
