# Supplementary material for: Novel MASP1 mutations are associated with an expanded phenotype in 3MC1 syndrome
Source: Orphanet J Rare Dis. 2015 Sep 30;10:128. doi: 10.1186/s13023-015-0345-3 (PMC4589207; doi:10.1186/s13023-015-0345-3)
Supplement: Additional file 1: Figure S1. — Pedigrees of the studied families. Figure S2. Electropherograms showing detected pathogenic variants. (DOCX 157 kb) [file 13023_2015_345_MOESM1_ESM.docx]

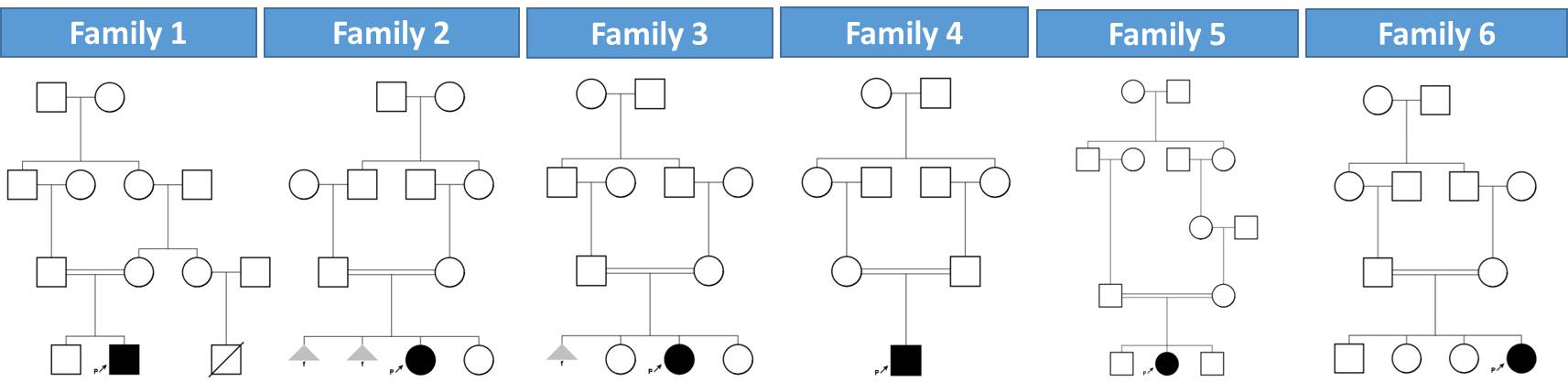


Supplemental Fig.1. Pedigrees of the studied families.


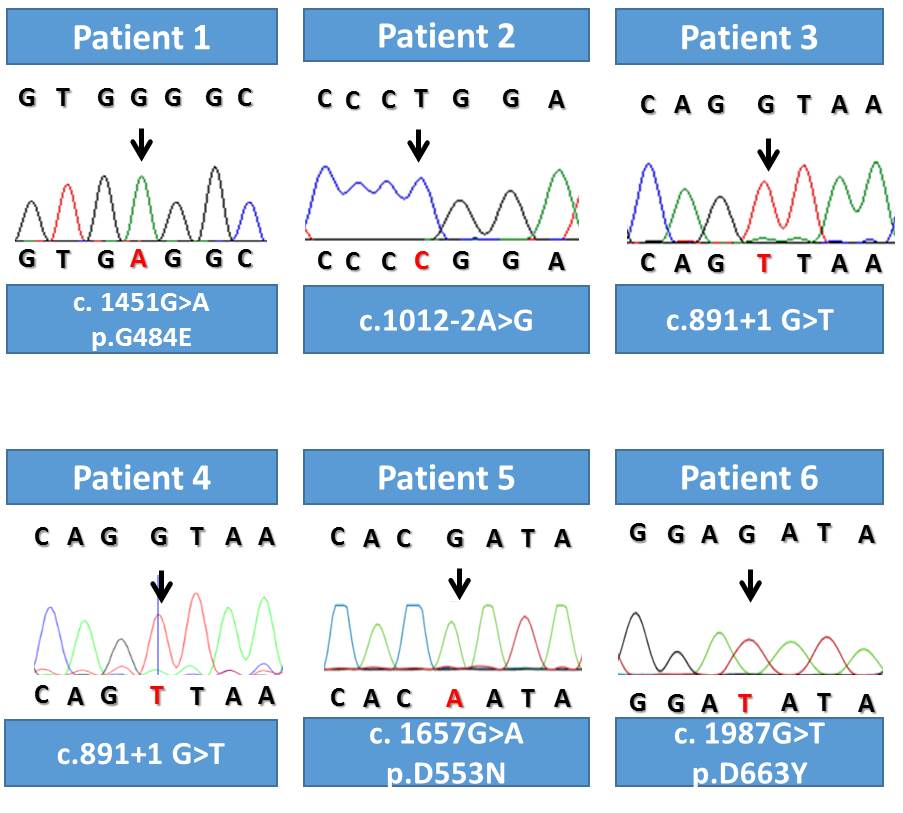


Supplemental Fig.2. Electropherograms showing detected pathogenic variants.
